# Hypoxia-Inducible Factor 2-Dependent Pathways Driving Von Hippel–Lindau-Deficient Renal Cancer

**DOI:** 10.3389/fonc.2018.00214

**Published:** 2018-06-08

**Authors:** Florinda Meléndez-Rodríguez, Olga Roche, Ricardo Sanchez-Prieto, Julian Aragones

**Affiliations:** ^1^Research Unit, Hospital of Santa Cristina, Research Institute Princesa (IP), Autonomous University of Madrid, Madrid, Spain; ^2^CIBER de Enfermedades Cardiovasculares, Carlos III Health Institute, Madrid, Spain; ^3^Unidad Asociada de Biomedicina, Universidad de Castilla-La Mancha, Consejo Superior de Investigaciones Científicas (CSIC), Albacete, Spain; ^4^Departamento de Ciencias Médicas, Facultad de Medicina, Universidad Castilla-La Mancha, Albacete, Spain; ^5^Laboratorio de Oncología Molecular, Unidad de Medicina Molecular, Centro Regional de Investigaciones Biomédicas, Universidad de Castilla-La Mancha, Albacete, Spain; ^6^Departamento de Biología del Cáncer, Instituto de investigaciones Biomedicas Alberto Sols, Universidad Autónoma de Madrid, Consejo Superior de Investigaciones Científicas (CSIC), Madrid, Spain

**Keywords:** Von Hippel–Lindau, hypoxia-inducible factors, hypoxia-inducible factor 2, kidney, renal cancer, clear cell renal cell carcinoma

## Abstract

The most common type of the renal cancers detected in humans is clear cell renal cell carcinomas (ccRCCs). These tumors are usually initiated by biallelic gene inactivation of the Von Hippel–Lindau (VHL) factor in the renal epithelium, which deregulates the hypoxia-inducible factors (HIFs) HIF1α and HIF2α, and provokes their constitutive activation irrespective of the cellular oxygen availability. While HIF1α can act as a ccRCC tumor suppressor, HIF2α has emerged as the key HIF isoform that is essential for ccRCC tumor progression. Indeed, preclinical and clinical data have shown that pharmacological inhibitors of HIF2α can efficiently combat ccRCC growth. In this review, we discuss the molecular basis underlying the oncogenic potential of HIF2α in ccRCC by focusing on those pathways primarily controlled by HIF2α that are thought to influence the progression of these tumors.

## Introduction

Kidney cancer accounts for ~3% of all cancer diagnoses worldwide and most of them are classified as clear cell renal cell carcinomas (ccRCCs). The clear appearance of these tumor cells is the result of the intracellular lipid depositions (1–4). ccRCCs are initiated by biallelic gene inactivation of the Von Hippel–Lindau (VHL) factor in the renal epithelium. VHL is a component of the E3 ligase ubiquitin machinery essential for the regulation of hypoxia-inducible factors (HIFs). HIFs are transcription factors comprised of one HIFα subunit (HIF1α, HIF2α, or HIF3α) and a member of the HIFβ family also known as aryl hydrocarbon receptor nuclear translocator (ARNT). HIFβ subunits are stable but the stability of HIFα subunits is controlled by cellular oxygen availability through the prolyl 2-oxoglutarate-dependent Fe^2+^-dioxygenases PHD1, PHD2, and PHD3 (5, 6). In normoxic conditions, PHDs use oxygen to hydroxylate two conserved proline residues in the HIFα subunits. These hydroxylated prolyl residues can be recognized by the VHL/E3 ubiquitin ligase complex, leading to the proteasomal degradation of HIFα subunits (7, 8). However, in hypoxic conditions there is insufficient oxygen for PHDs to hydroxylate the HIFα subunits, precluding their recognition by VHL and leading to HIFα subunits stabilization and activation of an HIF-dependent transcriptional program (9–12). Thus, the loss of VHL in ccRCC leads to constitutive activity of HIFs in normoxic conditions and its transcriptional program, which are ultimately key determinants in the progression of ccRCC.

## Contrasting Properties of HIF1α and HIF2α in ccRCC

**Figure 1:**
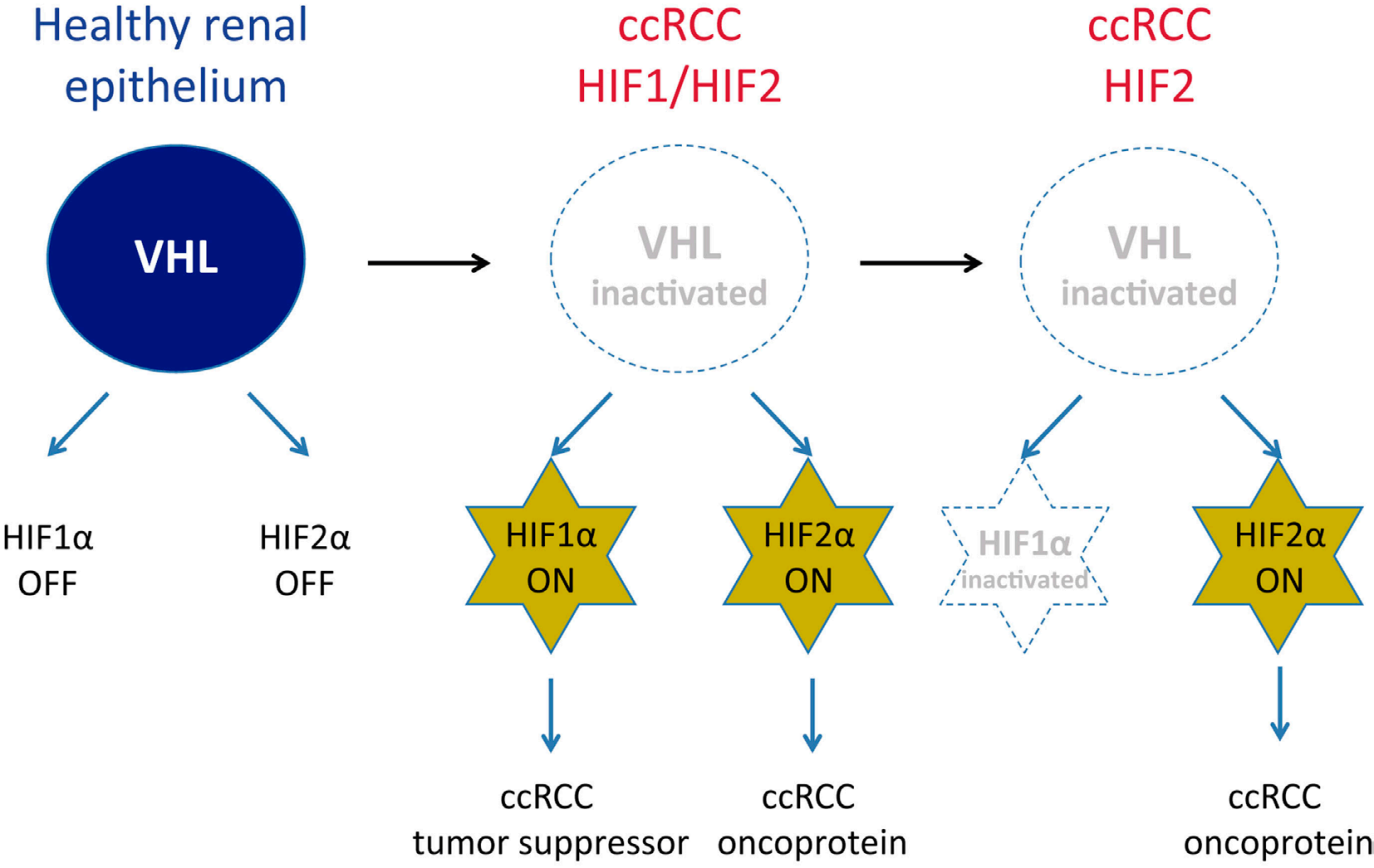
Expression of hypoxia-inducible factors (HIF)1α and HIF2α in Von Hippel–Lindau (VHL)-deficient clear cell renal cell carcinoma (ccRCC). The expression of VHL/E3 ubiquitin ligase complex leads to the proteasomal degradation of HIFα subunits, which assure that HIF1α and HIF2α inactivation in healthy renal epithelial cells. Upon VHL gene inactivation in ccRCC, HIF1α and HIF2α cannot be degraded and, therefore, are constitutively expressed in a large number of ccRCC. However, HIF1α acts as a ccRCC tumor suppressor and in this line, HIF1α locus is inactivated in some ccRCC while the expression of HIF2α—acting as a ccRCC oncoprotein—persists in some other ccRCC subtypes.

Initial somatic inactivation of the *VHL* gene in precancerous renal tubule lesions leads to HIF1α activation (13), as well as a progressive gain in HIF1α and HIF2α expression in dysplastic and cystic lesions (13). Moreover, mouse models of ccRCC have also shown that renal epithelium-specific HIF1α or HIF2α gene inactivation impairs ccRCC formation (14–16), indicating that both HIF1α and HIF2α are involved in ccRCC initiation. However, HIF1α expression is lost in 30–40% of overt ccRCCs, since HIF1α acts as a tumor suppressor during further progression of ccRCC by attenuating autonomous VHL-deficient tumor cell proliferation (Figure 1). Conversely, HIF2α acts as an oncoprotein in ccRCC (17–19). Therefore, overt ccRCC can be subdivided into those cases where both HIF1α and HIF2α are expressed, and those that only show HIF2α expression characterized by enhanced ccRCC cell proliferation and adverse prognosis (Figure 1) (17, 20–22). Therefore, the oncoprotein potential of HIF2α in ccRCC has led to the development of the HIF2α antagonists PT2399 and PT2385 to combat the progression of these tumors (23, 24). These HIF2α antagonists show inhibitory effects *in vivo* than those of the tyrosine kinase inhibitor sunitinib, which is used as a standard first-line therapy for metastatic ccRCC. In addition, PT2385 also appears to improve disease control in a patient who had been administered prior with other pharmacological therapies for ccRCC (23). In this review, we will focus on the cell autonomous pathways primarily controlled by HIF2α that have been shown to contribute to ccRCC progression.

## General Considerations About HIF2α-Dependent ccRCC Development

The protumoral potential of HIF2α in ccRCC have been studied extensively in VHL-deficient cell lines that express only HIF2α, such as the 786-O and A498 (24, 25) or those expressing both isoforms such as RCC4 cells (17, 26). Genetic or pharmacological inhibition of HIF2α usually in 786-O cells impairs their ability to form xenografts in nude mice and to generate colonies in soft agar conditions (18, 20, 25, 27, 28). However, such HIF2α inhibition does not alter the cell autonomous proliferation of these cells when they are grown in a petri dish under standard culture conditions (26–30). In RCC4 cells, inhibition of HIF2α can attenuate their normoxic *in vitro* cell proliferation under standard culture conditions (17, 26) although the extent of this effect is much less pronounced when compared with HIF2α inhibition *in vivo* in 786-O cells. Therefore, though the pro-proliferative properties of HIF2α in ccRCC can be appreciated in some cell culture conditions, they appear to be best observed when cells are subjected to experimental conditions that better mimic the three-dimensional solid tumor *in vivo*, such as xenografts in immunocompetent mice or colonies grown in soft agar.

**Figure 2:**
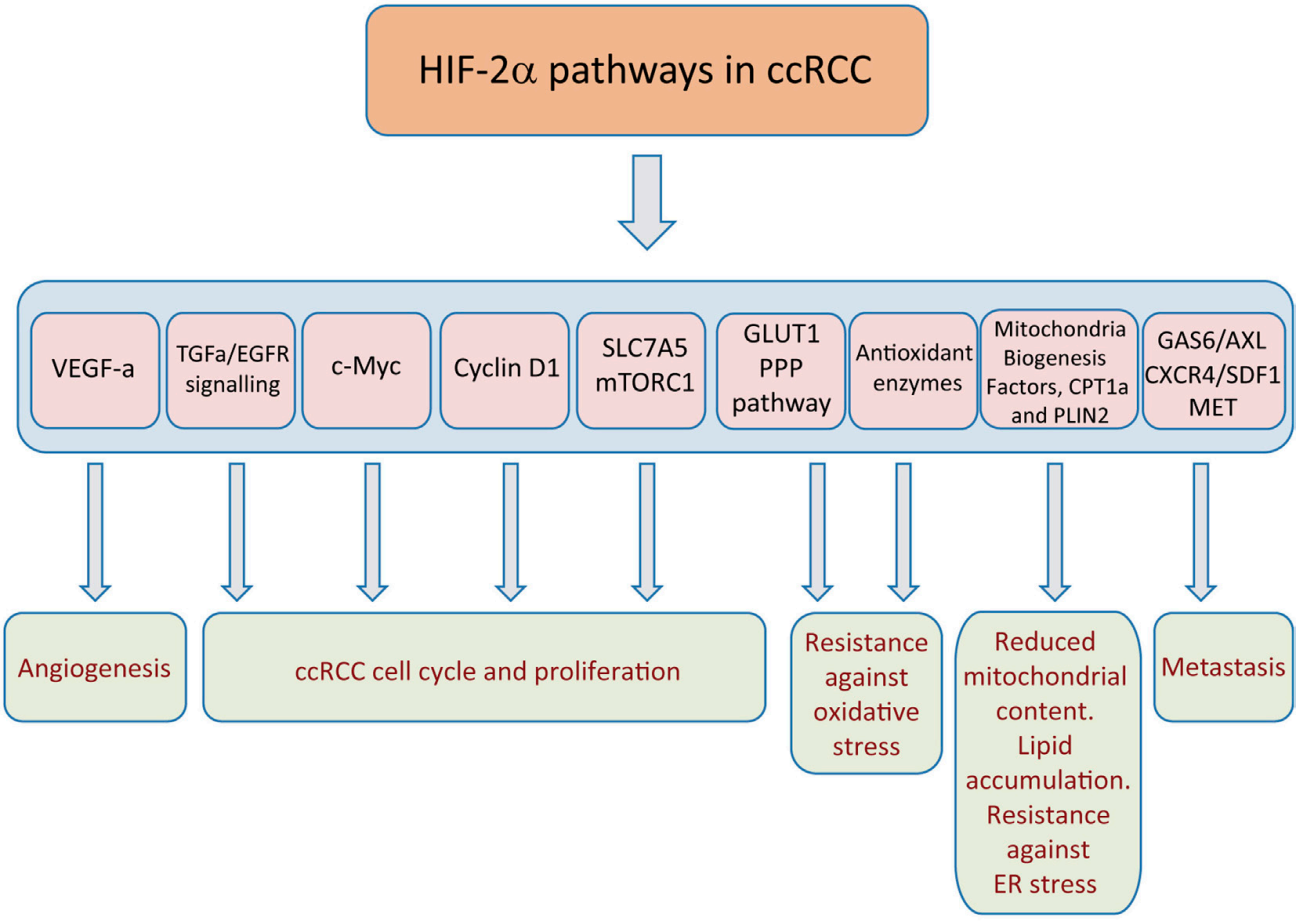
Hypoxia-inducible factor (HIF)2α-dependent pathways that sustain clear cell renal cell carcinoma (ccRCC) growth. The figure represents those target genes primarily controlled by HIF2α and those biological actions executed by these genes, such as tumor angiogenesis, cell autonomous proliferation, potentiation of glycolysis and pentose phosphate pathway (PPP), resistance to oxidative damage, endoplasmic reticulum (ER) stress, as well as metastasis.

A well-known target of HIF2α in ccRCC is vascular endothelial growth factor-a (VEGF-a), which drives ccRCC angiogenesis (23, 24, 31, 32). This HIF2/VEGF-a pathway does not seem to be required for *in vitro* 786-O cell proliferation but rather, it is essential for 786-O xenograft formation where intratumoral neoangiogenesis may be more critical (28). As such, this HIF2/VEGF-a pathway can explain the more pronounced impact of HIF2α on *in vivo* xenograft growth as opposed to *in vitro* cell proliferation (Figure 2). In this line, several pharmacological interventions to block ccRCC angiogenesis that target VEGF receptor (VEGFR) activity have shown clinical efficacy, such as sorafenib, pazopanib, or sunitinib (33).

## HIF2α-Dependent Cell Autonomous Pathways in ccRCC Growth

Hypoxia-inducible factor 2α-driven angiogenesis is certainly relevant to the progress of ccRCC, affecting distant vascular cells in a non-cell autonomous manner. However, from here on we will focus on other key HIF2α dependent, cell autonomous mechanisms that are critical to not only explain the protumoral properties of HIF2α in ccRCC but also the more remarkable effect of HIF2α in *in vivo* settings that are relevant to investigate ccRCC progression.

### HIF2α- and EGFR-Dependent Pathway

Regarding the effect of HIF2α in *in vivo* settings, it can be considered that cells in xenografts have less intratumoral access to nutrients or growth factors when compared with cells grown in standard monolayer cultures where nutrients and growth factors are more accessible. Indeed, the pro-proliferative potential of HIF2α on 786-O cells can best be observed when these cells are cultured in the low serum conditions (30). At the molecular level, HIF2α induces TGF-α expression, which in turns activates epidermal growth factor receptor (EGFR)-dependent cell signaling and proliferation (Figure 2) (30, 32). Moreover, it has been also shown that EGFR can be also transcriptionally regulated by HIF signaling (34) where HIF2α could also participate in its regulation in ccRCC. Interestingly, HIF2α can facilitate EGFR expression not only through transcriptional mechanisms but also by promoting EGFR mRNA translation. Indeed, the 3’ UTR region of EGFR mRNA contains an RNA binding site for HIF2α (rHRE) and the binding of HIF2α to this site accelerates EGFR mRNA translation. HIF2α forms a complex with the RNA-binding protein RBM4 and the cap-binding eIF4E2, an eIF4E homolog, which binds to the rHRE of EGRF mRNA and targets EGFR transcript to polysomes for active translation (35). Interestingly, this HIF2α–RBM4 complex has also been detected in ccRCC cells lines, irrespective of the oxygen tension (35), suggesting a possible role of this mechanism in ccRCC growth. Furthermore, HIF2α can also attenuate EGFR endocytosis, therefore, facilitating EGFR-dependent signaling in ccRCC cells (36). Moreover, HIF2α overexpression increases EGFR protein levels in 786-O cells (36). However, an independent study have shown that silencing endogenous HIF2α in 786-O does not alter EGFR protein levels in these cells (37).

Regarding the functional role of EGFR in ccRCC, silencing of EGFR signaling suppresses the ability of VHL-deficient 786-O cell lines to form xenografts. Along this line, it is also important to highlight that EGFR upregulation has been found in human ccRCC (34, 38, 39), which correlates with poor prognostic parameters (38). An additional study has also shown elevated EGFR expression in papillary RCC that is not characterized by HIF overactivation but in a lesser extent than in ccRCC (39). Furthermore, the expression of HCRP1, a repressor of EGFR signaling, is dampened significantly in ccRCC (40). In addition, it has also been found that the cell surface glycoprotein CUB domain-containing protein 1 (CDCP1) is induced through HIF2α—but not HIF1α—in response to hypoxia (41). EGF and CDCP1 cooperate to EGF-dependent intracellular signaling (42, 43), which suggest that this cooperation also occurs in ccRCC biology. In this line, CDCP1 is elevated in ccRCC and poor overall survival is found in patients with high CDCP1 expression (41).

In addition to EGFR function, HIF2α also potentiates the activity of the met proto-oncogene (MET) receptor (44–46). In this line, another tyrosin-kinase receptor AXL that is highly expressed in aggressive ccRCC tumors and associated with poor outcome has been shown to be a direct target gene of HIF2α but not HIF1α isoform (47). Indeed, GAS6/AXL signaling activates the met proto-oncogene (MET) receptor in an HGF-independent manner to optimize ccRCC migration and invasion without affecting primary tumor growth assessed using SN12L1 renal cancer cells (Figure 2) (47). Furthermore, HIF2α induces the expression of CXCR4 receptor, which facilitates the ability of its ligand—stromal cell-derived factor-1a (SDF-1a)—to promote ccRCC chemotaxis and influence patient survival (48–50).

### Role of HIF2α in the Regulation of c-Myc, Cell Cycle Regulators and p53

A key event in the molecular biology of ccRCC is the activation of c-Myc, a pro-proliferative transcription factor (Figure 2) (51, 52). In addition, in ccRCC, HIF1α and HIF2α have opposite effects on some c-Myc target genes that are involved in the cell cycle, providing a molecular basis for the opposite properties of both these HIF isoforms during overt ccRCC progression. Indeed, HIF1α impairs the binding of c-Myc to the regulatory regions of genes involved in the cell cycle, reducing E2F and cyclin D2 expression, while augmenting the expression of the cell cycle blockers p21 and p27 (21, 51). In sharp contrast, the HIF2α isoform potentiates c-Myc activity on these genes, regulating gene expression to favor cell cycle progression of ccRCC cells (Figure 2) (21, 51). Furthermore, this HIF2α-c-Myc pathway controls the expression of key effectors of homologous recombination or spindle assembly checkpoint, which limit DNA damage during replication (21).

Regarding the role of HIF2α in regulating the cell cycle machinery, cyclin D1 is another HIF2α target found specifically in renal cancers (Figure 2) (19). Indeed, a genetic variant at chromosome 11q13.3 is associated with a predisposition to renal cancer by permitting HIF2α binding to a cyclin D1 enhancer (53). Cyclin D1 is a cell cycle regulator involved in cancer cell proliferation and development (54), and its relative contribution to ccRCC has recently been evaluated in cyclin D1-silenced 786-O cells. Silencing cyclin D1 in 786-O cells does not alter their *in vitro* cell growth, but it does markedly attenuate their ability to form xenografts (28). Hence, like other HIF2α-dependent genes described in this review, cyclin D1 appears to be specifically required to sustain ccRCC growth *in vivo* without having a major impact on *in vitro* cell culture conditions.

Finally, HIF2α also prevents the activity of the tumor suppressor p53 to favor ccRCC survival and protect ccRCC cells from radiation treatment (20). Indeed, HIF2α induces the expression of several antioxidant enzymes in ccRCC, which restrict the oxidative stress-dependent p53 activation (Figure 2) (20). Therefore, inhibition of HIF2α permits the accumulation of reactive oxygen species (ROS) and DNA damage, leading to apoptosis and reduced survival of ccRCC cells (20).

### HIF2α and mTORC1 Activity in ccRCC

The mammalian target of rapamycin (mTOR) is a serine/threonine kinase that responds to amino acid availability, as well as energy status of the cell, playing a central role in cell growth and proliferation (55, 56). mTORC1 activation is required for ccRCC progression (57) and indeed, the allosteric inhibitors of mTORC1, everolimus, and temsirolimus, counteract ccRCC (58, 59). Such mTORC1 activation is also observed in mouse models of ccRCC in which *Vhl* and *Pten*, or *Vhl* and *Pbrm1*, are inactivated simultaneously in the epithelial cells of the genital tract (60, 61). This mTORC1 overactivation is a consequence of excess HIF2α activity in ccRCC (29). It should be considered first that mTORC1 responds to the availability of essential extracellular amino acids and second that amino acid supply presumably becomes compromised at the inner core of solid tumors (29, 62, 63). Along similar lines, HIF2α provides both an *in vitro* growth advantage to ccRCC cells as well as a potentiation of mTORC1 activity when cells are exposed to low amino acid supply, which to some extent could mimic the limited intratumoral amino acid availability (29).

At the molecular level, HIF2α induces the expression of the amino acid carrier SLC7A5 (LAT-1), which is essential to promote amino acid-dependent mTORC1 activation, and to sustain the potential of 786-O cells to form xenografts in nude mice (Figure 2) (29, 64). Enhanced SLC7A5 expression is not only found in 786-O VHL-deficient cells but also, in human ccRCC samples. Importantly, SLC7A5 protein is markedly elevated in VHL-deficient ccRCC samples—but not in non-clear renal cell carcinomas (ncRCC)—when compared with adjacent healthy kidney (29). An independent study has also confirmed this SLC7A5 elevation at RNA level in VHL-deficient ccRCC when compared with non-tumor samples (65). Importantly, increased SLC7A5 expression favors amino acid-dependent mTORC1 activity and cell proliferation, preferentially in low amino acid conditions (29, 66, 67). The relevance of SLC7A5 in low amino acid conditions can also explain the marked effect of HIF2α in *in vivo* ccRCC settings, when a limited intratumoral amino acid supply would be expected. Furthermore, the influence of HIF2α on SLC7A5 expression is not restricted to renal cell carcinoma and it has actually been found in other biological settings, such as cervical cancer cells (68) as well as in other non-tumoral scenarios (29, 69).

Paradoxically, HIF2α also increases the expression of REDD1, a well-recognized mTORC1 inhibitor (70). REDD1 is not capable of repressing mTORC1 in 786-O cells but this is not generalized, since it does not occur in other VHL-deficient cell lines (71). Therefore, in ccRCC a HIF2α-dependent mTORC1 activation pathway overrides the mTORC1 inhibitory potential of REDD1 (71). Indeed, it is likely that elevated REDD1 in ccRCC could limit full mTORC1 activation but not potent enough to prevent HIF2α-dependent mTORC1 activated pathways.

### HIF2α and ccRCC Glucose and Lipid Metabolism

A central metabolic response to HIF activation is an anaerobic switch favoring glycolysis, while simultaneously repressing mitochondrial function (72–74). The HIF-dependent regulation of glycolysis is mainly executed by the HIF1α primarily inducing the vast majority of glycolytic enzymes (75–77). However, HIF2α is also a potent inducer of glucose transporter-1 (GLUT-1) (18, 19) and of enolase 2 (ENO2) (28) in ccRCC (Figure 2), which also anticipates an increased rate of glycolysis in cells expressing only HIF2α. Glycolytic intermediates corresponding to the first steps of the glycolytic pathway that are required to foster the pentose phosphate pathway are markedly elevated in human ccRCC, possibly contributing to counteract oxidative stress (Figure 2) (78–80). Regarding the relative contribution of GLUT-1 to ccRCC biology, VHL-deficient RCC4 cells are particularly sensitive to glucose deprivation and *GLUT-1* silencing provokes apoptosis of these cells (81). Furthermore, pharmacological inhibition of GLUT-1 with STF-31 selectively kills VHL-deficient cells and attenuates the ability of 786-O cells to form xenografts (81). Surprisingly, an independent study has shown that silencing of GLUT-1 or ENO-2 does not prevent these 786-O cells from generating xenografts (28). The reasons for this discrepancy are unclear and possibly reflect differences between the pharmacological and genetic approaches to assure GLUT-1 inhibition in 786-O cells. Despite of this contrasting information, ccRCC are characterized by accumulation of glycolytic intermediates as mentioned above in line with GLUT-1 elevation. Furthermore, HIF2α not only increases GLUT-1 expression, probably to increase in the glycolytic rate but also, it simultaneously attenuates glucose oxidation in parallel with an increase in glutamine usage *via* the reductive carboxylation pathway (82). These metabolic actions seem to be executed even in the absence of HIF1α isoform, which has been more widely recognized to be essential to attenuate glucose oxidation and promote glutamine reductive carboxylation (83–85). Indeed, 786-O cells use reductive glutamine metabolism to generate the citrate that is essential to sustain lipogenic pathways that are required for cell proliferation (82).

In addition to this glucose and glutamine metabolism reprogramming, a key metabolic feature that defines ccRCC is the remarkable accumulation of intracellular lipid droplets (86). These lipid droplets are the sites where cells store neutral lipids, such as triglycerides, steryl esters, and retinyl esters that are surrounded by a phospholipid monolayer including associated lipid droplets surface proteins (87). HIF2α—together with HIF1α—participates in the lipid deposition in ccRCC. First, HIF2α drives lipid deposition in ccRCC by repressing fatty acid oxidation, specifically the rate-limiting component of mitochondrial fatty acid transport, carnitine palmitoyltransferase 1A (CPT1A) (88). Second, HIF2α can reduce mitochondrial content as well as key factors in mitochondrial biogenesis that also characterized VHL-deficient renal cell carcinoma cells (89–91) which can also explain not only reduced fatty acid oxidation but also glucose oxidation in ccRCC cells. Importantly, HIF2α, but not HIF1α, also controls the expression of Perilipin 2 (PLIN2) (Figure 2), a lipid droplet coat protein that regulates lipid storage and lipolysis (32). These droplets are functionally and physically associated with the endoplasmic reticulum (ER), and, therefore, this HIF2α-PLIN2-dependent lipid droplet formation not only favors the accumulation of excess fatty acids possibly as a consequence of reduced fatty acid oxidation but also avoids cytotoxic ER stress which facilitates the progression of ccRCC (Figure 2).

## Conclusion

Hypoxia-inducible factor 2α drives numerous pathways that favor ccRCC proliferation and survival of these tumors. Importantly, these pathways seem to be specially relevant under *in vivo* conditions, where tumor mass can be characterized by compromised oxygen supply, nutrient availability (e.g., glucose or amino acids), possibly less access to growth factors, such as EGFR ligands, as well as stressful intratumoral conditions. In this line, HIF2α and their pathways seem less relevant when ccRCC cells are grown under *in vitro* monolayer conditions, where nutrient, oxygen supply and growth factors supply can be unlimited. In this line, HIF2α favors simultaneously the expression of GLUT-1 glucose transporter, SLC7A5 amino acid carrier, as well as VEGFa-dependent angiogenesis, which all together can favor ccRCC nutrient and oxygen supply *in vivo*. Moreover, HIF2α also potentiates EGFR signaling and promotes signals that alleviate oxidative and ER stress promoting ccRCC survival. Along this line, current therapies that focus on HIF2α inhibition have the potential to concomitantly affect all the pathways described here, without the need to focus on each individual pathway described here.

## Author Contributions

JL wrote the manuscript and designs the general structure, sections, and topics to be discussed in this mini-review. FM-R, OR, and RSP wrote specific parts of this mini-review.

## Conflict of Interest Statement

The authors declare that the research was conducted in the absence of any commercial or financial relationships that could be construed as a potential conflict of interest.
